# A novel germline *POLE* mutation causes an early onset cancer prone syndrome mimicking constitutional mismatch repair deficiency

**DOI:** 10.1007/s10689-016-9925-1

**Published:** 2016-08-29

**Authors:** Katharina Wimmer, Andreas Beilken, Rainer Nustede, Tim Ripperger, Britta Lamottke, Benno Ure, Diana Steinmann, Tanja Reineke-Plaass, Ulrich Lehmann, Johannes Zschocke, Laura Valle, Christine Fauth, Christian P. Kratz

**Affiliations:** 1 Division of Human Genetics, Medical University Innsbruck, Peter-Mayr-Straße 1, 6020 Innsbruck, Austria; 2 Pediatric Hematology and Oncology, Hannover Medical School, Hannover, Germany; 3 Department of Surgery, Children’s Hospital, Hannover Medical School, Hannover, Germany; 4 Institute of Human Genetics, Hannover Medical School, Hannover, Germany; 5 Department of Radiotherapy and Special Oncology, Hannover Medical School, Hannover, Germany; 6 Institute of Pathology, Hannover Medical School, Hannover, Germany; 7 Hereditary Cancer Program, Catalan Institute of Oncology, IDIBELL, Hospitalet De Llobregat, Spain

**Keywords:** Polymerase proofreading-associated polyposis, Constitutional mismatch repair deficiency, Café-au-lait macule, Pilomatricoma, Colon cancer

## Abstract

In a 14-year-old boy with polyposis and rectosigmoid carcinoma, we identified a novel *POLE* germline mutation, p.(Val411Leu), previously found as recurrent somatic mutation in ‘ultramutated’ sporadic cancers. This is the youngest reported cancer patient with polymerase proofreading-associated polyposis indicating that *POLE* mutation p.(Val411Leu) may confer a more severe phenotype than previously reported *POLE* and *POLD1* germline mutations. The patient had multiple café-au-lait macules and a pilomatricoma mimicking the clinical phenotype of constitutional mismatch repair deficiency. We hypothesize that these skin features may be common to different types of constitutional DNA repair defects associated with polyposis and early-onset cancer.

## Introduction

Faithful DNA replication of human cells is safeguarded by two mechanisms: (1) proofreading by an exonuclease activity intrinsic to the replication polymerases, and (2) DNA mismatch repair (MMR) [1]. Constitutional inactivation of either mechanism causes cancer susceptibility syndromes.

Polymerase proofreading-associated polyposis (PPAP, MIM 612591 and 615083) results from heterozygous missense *POLD1* and *POLE* exonuclease domain mutations (EDMs) inactivating the proofreading activity of the replicative polymerases, Pol δ and Pol ε, respectively [2]. One recurrent *POLE* mutation, p.(Leu424Val), and five *POLD1* mutations, p.(Ser478Asn), p.(Leu474Pro), p.(Asp316His), p.(Asp316Gly), p.(Asp316Gly), and p.(Arg409Trp), account for so far 21 and 8 families with PPAP, respectively [3]. The median age at first cancer diagnosis was 40 years (range: 27–64 years) in 30 CRC patients carrying the *POLE* mutation and 32.5 years (range 23–57 years) in 13 CRC patients with *POLD1* mutations [3].

Constitutional MMR deficiency syndrome (CMMRD, MIM 276300) is caused by biallelic germline mutations in one of the four mismatch repair (MMR) genes, i.e. *MLH1*, *MSH2*, *MSH6*, and *PMS2* [4]. CMMRD confers a more severe phenotype with a high childhood cancer risk. The CMMRD tumor spectrum is broad and includes hematological malignancies and brain tumors [5]. Most patients, who reach adolescence, will develop colorectal adenomas, which rapidly transform into carcinomas [6]. In addition, CMMRD manifests with non-malignant features, which serve as diagnostic signposts for this recessively inherited childhood cancer syndrome [7]. The most prevalent features are multiple café-au-lait macules (CALMs) and other pigmentary alterations. Moreover, patients with CMMRD commonly develop typically asymptomatic slow growing benign skin tumors termed pilomatricomas (pilomatrixoma, calcifying epithelioma of Malherbe). Both, CALMs and pilomatricomas, serve as diagnostic features in a 3-point scoring system, recently established by the European consortium “Care for CMMRD” (C4CMMRD) [7]. According to this scoring system, any pediatric/young adult cancer patient reaching a minimum of 3 scoring points is suspected of having CMMRD.

## Subjects and methods

### Case report

**Fig. 1:**
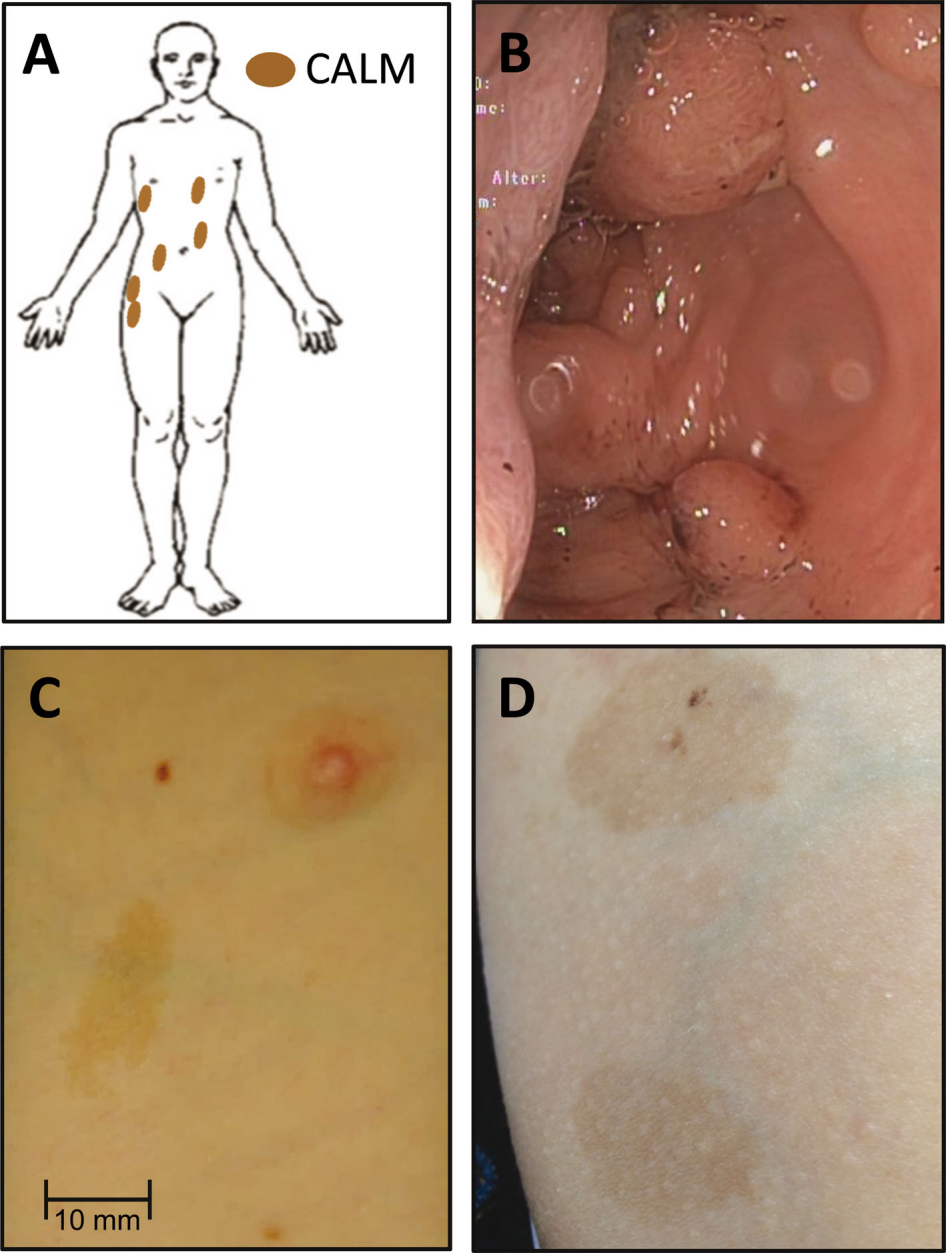
Clinical presentation of the patient. Body scheme showing the distribution of six café-au-lait macules (CALMs) (**a**). Colonoscopy image showing multiple polyps in a section of the left hemicolon (**b**). Representative CALMs from trunk, note the irregular (cost of Maine shaped) border (**c**) and right hip (**d**)

The index patient is a 14-year-old boy, who was referred from a secondary care hospital to the Department of Pediatric Hematology and Oncology of Hannover Medical School for treatment of colorectal cancer. The boy had a multi-locular mucinous rectosigmoid adenocarcinoma rendering three points in the CMMRD scoring system. He had a tubular duodenal adenoma and multiple (>10 larger and uncountable smaller) tubular and tubulovillous adenomas in the colon descendens, sigmoid, and rectum, of which some displayed high grade intraepithelial neoplasia (three points; Fig. 1b). In addition, multiple (n = 6) CALMs on trunk and right hip (two points; Fig. 1a, b, d) and one firm pea-sized nodule located over the left ear, clinically diagnosed as pilomatricoma (one point), were present. Taken together, these features sum-up to a total of nine points and, therefore, the diagnosis CMMRD was highly suspected. The non-consanguineous parents reported absence of Lynch syndrome-associated tumors in their families.

### Analysis of the tumor tissue

Immunohistochemical (IHC) analysis: IHC of MMR gene expression was performed on the paraffin-embedded rectosigmoid adenocarcinoma tissue according to standard laboratory procedures [8]. Monoclonal antibodies anti-MLH1 (G168-15, dilution 1:40, BD Biosciences, Heidelberg, Germany), anti-MSH2 (FE11, dilution 1:200, Calbiochem, Merck, Darmstadt, Germany), anti-MSH6 (44, dilution 1:400; BD Biosciences), anti-PMS2 (A16-4, dilution 1:200; BD Biosciences) and avidin–biotin–peroxidase complexes were used.

Microsatellite instability (MSI) analysis: Tumor and normal tissue were isolated by manual microdissection from unstained formalin-fixed and paraffin-embedded histological sections using an H&E-stained section as guidance. DNA was extracted using the DNeasy Blood and Tissue Kit (Qiagen, Hilden, Germany) following the manufacturer’s instructions. Subsequently, the following markers were PCR-amplified using fluorescence-labeled primers: BAT25, BAT26, APC, MFD15, and D2S123 [9, 10]. PCR products were separated by capillary electrophoresis using the GenomeLab GeXP Genetic Analysis System (Beckman Coulter, Krefeld, Germany). Fragment patterns were generated and analyzed using the instrument´s software.

### Germline analysis

Germline MSI (gMSI) analysis was performed according to the protocols developed by Ingham et al. [11]. In brief, dinucleotide microsatellite markers D2S123, D17S250, and D17S791 were PCR amplified from genomic DNA extracted from blood lymphocytes. Subsequently, PCR products were separated by capillary electrophoresis on an ABI3130 genetic analyzer. gMSI ratios are calculated using PeakHeight-Software (http://dna.leeds.ac.uk/peakheights/) and are compared to laboratory-specific threshold ratios, which are defined as the mean + 3-times standard deviation (SD) of 80–90 control DNAs. Germline microsatellite stability is stated if none of the three investigated microsatellite markers shows a gMSI ratio above the laboratory specific threshold.

Mutation analysis of the MMR genes *MLH1* (NM_000249.3), *MSH2* (NM_000251.2), *MSH6* (NM_000179.2), *PMS2* (NM_000535.5), and the polyposis syndromes associated genes *APC* (NM_00038.5), *MUTYH* (NM_1128425.1), *PTEN* (NM_000314.4), *STK11* (NM_000455.4), *BMPR1A* (NM_004329.2), and *SMAD4* (NM_005359.5) was performed by massive parallel sequencing analysis. The target sequences were enriched from genomic DNA extracted from blood lymphocytes by Nextera Rapid Capture technology using the pre-designed TruSight Cancer kit (Illumina San Diego, CA) according to the manufacturer’s protocols. Sequencing with reversible dye terminator was performed on a MiSeq instrument (Illumina). The NextGENe^®^ software (SoftGenetics, State College, USA) was used for sequence alignment to the human reference sequence GRCh37 (hg19) and variant calling. Variants of the target genes were displayed and evaluated in GeneticistAssistant™ (SoftGenetics). Analysis for copy number variations (CNV), i.e. deletions and duplications of one or more exons of the target genes, was performed with the dispersion based CNV tool included in the NextGENe^®^ software.

The exonuclease domains of *POLE* (NM_006231.3, exons 9–14) and *POLD1* (NM_002691.3, exons 6–12) were investigated for missense mutations by direct automated Sanger sequencing as previously described [3].

All variants are described in accordance with the recommendations of the Human Genome Variation Society (http://www.hgvs.org/mutnomen).

## Results

**Fig. 2:**
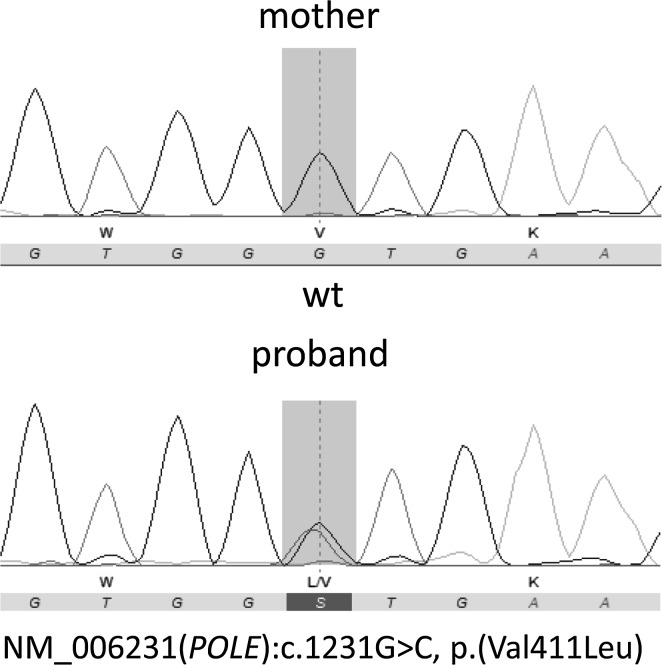
Sanger sequencing chromatograms showing the *POLE* genotype of the proband carrying the *POLE* mutation p.(Val411Leu) and his mother, in whom this mutation is absent

Extensive analysis was undertaken to evaluate the suspected diagnosis CMMRD in the index patient. Microsatellite instability (MSI) analysis revealed stability for all five investigated microsatellite markers in tumor tissue. IHC showed expression of all four MMR genes in neoplastic and non-neoplastic cells. No MSI was observed by gMSI testing according to the protocol developed by Ingham et al. [11]. Massive parallel sequencing-based molecular genetic analysis of all four MMR genes did not detect point mutations or copy number alterations. Thus, CMMRD could not be confirmed. Subsequent massive parallel sequencing-based mutation analysis of *APC*, *MUTYH*, *PTEN*, *STK11*, *BMPR1A,* and *SMAD4* also failed to reveal a germline mutation. Finally, Sanger sequencing of the entire exonuclease domains of *POLD1* and *POLE* uncovered the heterozygous *POLE* mutation NM_006231.3:c.1231G > C (p.Val411Leu) (Fig. 2). This mutation was not previously described as germline alteration, but is likely to be functionally relevant, because it is a somatic hot-spot mutation recurrently found in ‘ultramutated’ sporadic colorectal and endometrial cancers with a mutational profile characteristic for impaired Pol ε exonuclease activity [12]. Furthermore, ex vivo experiments showed that this mutation impairs the proofreading activity of Pol ε [12]. Therefore, this *POLE* mutation most likely is the cause of the polyposis and early onset rectosigmoid cancer in the patient. In agreement with the reported absence of Lynch syndrome-associated tumors in her family, the mutation was excluded in the healthy 36-year-old mother. The healthy 43-year-old father was not available for genetic testing.

## Discussion

This 14-year-old boy is by far the youngest reported PPAP patient with carcinoma and extensive polyposis. Hence, although cell-free experiments show comparable effects of the somatic hotspot *POLE* mutation p.(Val411Leu) and the recurrent germline mutation p.(Leu424Val) on exonuclease activity [12], the detection of p.(Val411Leu) as a constitutional mutation in this 14-year-old cancer patient suggests that this *POLE* mutation confers a more severe phenotype than the previously reported *POLE* and *POLD1* germline mutations [3]. Differences in the mechanism of action of p.(Val411Leu) and the more frequent *POLE* and *POLD1* germline mutations are not fully understood, but it is noteworthy that in contrast to *POLE* residue p.Leu424 and to the residues affected by the key *POLD1* mutations, residue p.Val411 lies some distance away from the exonuclease catalytic site [13]. Therefore, mutation p.(Val411Leu) is thought to act through secondary effects on the exonuclease activity [13], which may influence the clinical phenotype. Currently, however, it cannot be excluded that other modifying variants influenced the phenotype of our patient.

Not only the adolescence-onset of CRC, but also the multiple CALMs and the pilomatricoma in our patient are reminiscent of CMMRD. It is speculated that these features occur more frequently in CMMRD patients than in the general population, because they result from post-zygotic mutations in genes prone to somatic mutation in individuals with impaired MMR capacity. In support of this notion, it was shown that distinct pilomatricomas occurring in a single CMMRD patient resulted from different activating *CTNNB1* (ß-Catenin gene) mutations. This suggests that certain genes may be targeted repeatedly by independent somatic mutational events in CMMRD patients [14]. Accordingly, the constitutional polymerase proofreading defect may explain the occurrence of these features in our patient. We hypothesize that a skin phenotype characterized by pigmentation alterations and/or pilomatricoma formation in childhood and adolescence might be common to and indicative of a constitutional defect in the repair of replication errors.

Apart from PPAP and CMMRD there is a growing number of other cancer syndromes primarily associated with polyposis and CRC that result from constitutional DNA repair defects. *MUTYH*-associated polyposis (MAP, MIM 608456) and the recently delineated *NTHL1*-associated polyposis (NAP, MIM 616415) are caused by biallelic germline mutations inactivating the base excision repair genes *MUTYH* [15], and *NTHL1* [16, 17], respectively. Adam et al. [18] showed in two unrelated families that biallelic truncating mutations in the fifth MMR gene, *MSH3,* cause a similar polyposis syndrome. Of note, multiple pilomatricomas were also reported in two siblings with MAP due to a homozygous truncating *MUTYH* mutation [19]. Therefore, the delineated skin phenotype may be also indicative for constitutional defects in base excision repair. To corroborate this notion, systematic investigations for CALMs and pilomatricomas need to be conducted in patients with constitutional defects not only in replication error but also in base excision repair.

In conclusion, this case shows that PPAP is a differential diagnosis in patients presenting with polyposis and/or colorectal cancer and skin phenotype indicative of CMMRD. Since high grade glioma (HGG), reported so far in 2/47 patients with PPAP [3], may belong to the tumor spectrum of PPAP, this diagnosis should also be considered in pediatric and young adult patients with HGG (and possibly other cancers), who show signs reminiscent of CMMRD but lack diagnostic hallmarks of CMMRD, such as MMR protein expression loss and other diagnostic features [20], and/or biallelic MMR gene mutations. The identification of further PPAP patients will clarify whether only specific exonuclease domain mutations confer a phenotype reminiscent of CMMRD.
